# External Validation of the Extraprostatic Extension Grade on MRI and Its Incremental Value to Clinical Models for Assessing Extraprostatic Cancer

**DOI:** 10.3389/fonc.2021.655093

**Published:** 2021-04-01

**Authors:** Lili Xu, Gumuyang Zhang, Xiaoxiao Zhang, Xin Bai, Weigang Yan, Yu Xiao, Hao Sun, Zhengyu Jin

**Affiliations:** ^1^Department of Radiology, Peking Union Medical College Hospital, Peking Union Medical College, Chinese Academy of Medical Sciences, Beijing, China; ^2^Department of Urology, Peking Union Medical College Hospital, Peking Union Medical College, Chinese Academy of Medical Sciences, Beijing, China; ^3^Department of Pathology, Peking Union Medical College Hospital, Peking Union Medical College, Chinese Academy of Medical Sciences, Beijing, China

**Keywords:** prostatic neoplasms, magnetic resonance imaging, extraprostatic extension, risk assessment, pathological grade

## Abstract

**Objectives:**

To externally validate the extraprostatic extension (EPE) grade criteria on MRI and analyze the incremental value of EPE grade to clinical models of prostate cancer.

**Methods:**

A consecutive 130 patients who underwent preoperative prostate MRI followed by radical prostatectomy between January 2015 to January 2020 in our institution were retrospectively enrolled. The EPE grade, Cancer of the Prostate Risk Assessment (CAPRA), and Memorial Sloan Kettering Cancer Center nomogram (MSKCCn) score for each patient were assigned. Significant clinicopathological factors in univariate and multivariate analyses were combined with EPE grade to build the Clinical + EPE grade model, and the CAPRA and MSKCCn score were also combined with EPE grade to build the CAPRA + EPE grade and MSKCCn + EPE grade model, respectively. The area under the curve (AUC), sensitivity and specificity of these models were calculated to evaluate their diagnostic performance. Calibration and decision curve analyses were used to analyze their calibration performance and clinical utility.

**Results:**

The AUC for predicting EPE was 0.767–0.778 for EPE grade, 0.704 for CAPRA, and 0.723 for MSKCCn. After combination with EPE grade, the AUCs of these clinical models increased significantly than using clinical models along (*P* < 0.05), but was comparable with using EPE grade alone (*P* > 0.05). The calibration curves of EPE grade, clinical models and combined models showed that these models are well-calibrated for EPE. In the decision curve analysis, EPE grade showed slightly higher net benefit than MSKCCn and CAPRA.

**Conclusion:**

The EPE grade showed good performance for evaluating EPE in our cohort and possessed well clinical utility. Further combinations with the EPE grade could improve the diagnostic performance of clinical models.

## Introduction

Prostate cancer (PCa) is the most common malignancy in men worldwide (1). Extraprostatic extension (EPE) of PCa is associated with an increased risk of positive surgical margins (2), biochemical recurrence (3), and even death from PCa (4, 5). Preoperative prediction of EPE has an important influence on clinical decision making. Patients without EPE could consider nerve-sparing radical prostatectomy or active surveillance according to their risk stratification, while patients with positive EPE are recommended to undergo nerve-sacrificing radical prostatectomy or adjuvant radiotherapy (6, 7).

Previously, some clinical models and grading systems have been proposed for preoperative evaluation of EPE, including the Cancer of the Prostate Risk Assessment (CAPRA) score (8), Memorial Sloan Kettering Cancer Center nomogram (MSKCCn) (9), and Partin tables (PT) (10). These models are based on clinical and histopathological variables, such as prostate-specific antigen (PSA) level, biopsy Gleason score (GS), and clinical T stage. Nevertheless, the diagnostic performance of these models varies with reported areas under the curve (AUCs) ranging from 0.610 to 0.806 (9–12).

MRI is an important preoperative evaluation method for PCa, which has been reported to be useful for predicting EPE. Regarding the limitations of previous MRI criteria for EPE evaluation, there is heterogeneity in the definitions of positive and negative results and significant inter-reader variability (13). Mehralivand et al. proposed a standardized and more simplified MRI grading system (termed the EPE grade) for EPE evaluation (14). This EPE grading system showed comparable diagnostic performance with other MRI criteria, including the European Society of Urogenital Radiology score, capsular contact length, and Likert scales, and possessed the highest correlation with histologic EPE extent (15). Nevertheless, there is still a lack of a direct comparison of EPE grade with the presently existing clinical models, and the incremental value of the EPE grade to clinical variables remains unknown.

Therefore, this study was designed to externally validate the EPE grade, compare it with the MSKCCn and CAPRA score, and analyze whether combining the EPE grade with clinical variables and clinical models would improve their diagnostic performance.

## Materials and Methods

### Patients

**Figure 1:**
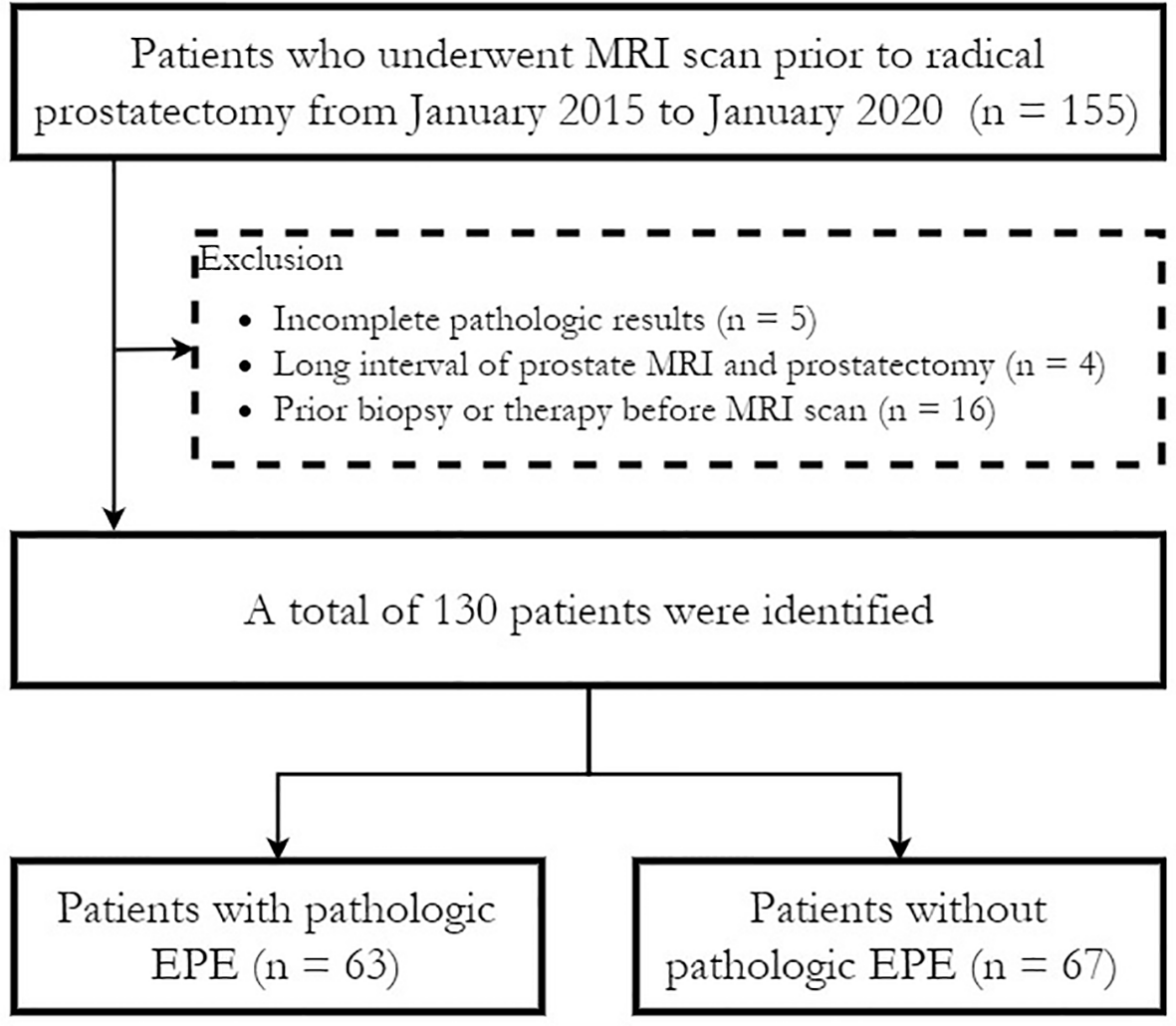
Flow diagram of patient selection for the study. EPE, extraprostatic extension.

The Institutional Review Board (IRB) approved this retrospective study (IRB number JS-2114) and waived the need for written informed consent. Consecutive patients with pathologically confirmed prostate cancer who underwent preoperative prostate multiparametric MRI (mpMRI) followed by radical prostatectomy between January 2015 to January 2020 in our institution were retrospectively enrolled in this study. The exclusion criteria were as follows (1): preoperative biopsy results were not available or complete pathological slices were not available for EPE evaluation (n = 5); (2) the interval between prostate MRI and radical prostatectomy was more than six months (n = 4); and (3) patients who received a biopsy (within 6 months before MRI), radiation therapy or hormonal therapy before MRI (n = 16). No patients received neo-adjuvant androgen deprivation therapy (ADT). **Figure 1** shows a flowchart of patient recruitment in this study, and a total of 130 patients were finally enrolled.

The clinicopathologic data including age, PSA level, clinical T stage, biopsy GS, biopsy International Society of Urological Pathology (ISUP) category, and percentage of positive biopsy cores for each patient were obtained from the medical records. According to the patients’ PSA level, GS, and clinical stage, patients were stratified into low-, intermediate-, and high-risk/locally advanced groups (16). Additionally, the CAPRA score (8) and MSKCCn score (17) for each patient were also calculated.

### MR Data Acquisition

A 3.0-T MRI scanner (GE750, GE Healthcare) was used to perform prostate mpMRI, including T2-weighted imaging (T2WI), diffusion-weighted imaging (DWI), and dynamic contrast-enhanced (DCE) imaging. Corresponding apparent diffusion coefficient (ADC) maps were calculated using b values of 0 and 800 mm^2^/sec. The detailed MR imaging acquisition parameters applied in this study are shown in **Supplementary Table 1**.

### Image Interpretation

Mehralivand et al.’s EPE grade imaging criteria (14) were used to assess EPE likelihood: grade 0, no suspicion for EPE; grade 1, either curvilinear contact length ≥ 1.5 cm or capsular irregularity and bulge; grade 2, both curvilinear contact length ≥ 1.5 cm and capsular irregularity and bulge; grade 3, frank EPE visible at MRI or invasion of adjacent anatomic structures. Since our Picture Archiving and Communication Systems (PACS) doesn’t contain a free-hand measurement tool, the curvilinear contact length was estimated by drawing a series of measurements (usually 2–3 straight lines). All examinations were interpreted by one senior radiologist (Reader 1, with 7 years of experience in interpreting prostate MRI, interpreted more than 2 000 cases) who was unaware of the presence or absence of pathologic EPE or clinical variables. When multiple lesions existed, the lesion with the highest EPE grade was enrolled for analysis. The lesion’s Prostate Imaging Reporting and Data System version 2.1 (PI-RADS v2.1) category and tumor location were also recorded. Prostate volume at MRI was calculated using the formula for a prolate ellipse: (maximum anterior–posterior diameter) × (maximum transverse diameter) × (maximum longitudinal diameter) × 0.52. PSA density (PSAD) = PSA/prostate volume.

Another radiologist (Reader 2, with 2 years of experience in interpreting prostate MRI, interpreted about 300 cases) also reviewed the images to calculate the interreader variability of this EPE grade. All mpMRI studies were re-evaluated by the same radiologist after 4 weeks to assess the intrareader agreement.

### Standard of References

The final histopathologic assessment was defined as the standard reference. One senior pathologist (with more than 10 years of experience in prostate specimen interpretation) who was blinded to the MRI reports reviewed the pathological slices (with a whole-mount slice thickness of 0.4 cm) and recorded the presence or absence of EPE for each patient. EPE was defined as the presence of prostate tumors extending out of the confines of the prostate (18). After image interpretation and pathology evaluation, another radiologist (Reader 3) performed the site concordance procedure, and matched the lesions evaluated on MRI with pathology specimen.

### Statistical Analysis

The differences in clinicopathological variables between the EPE positive and EPE negative groups were assessed using the Mann-Whitney *U* test, chi-squared test, or Fisher’s exact test, where appropriate. Subsequently, the forward stepwise logistic regression method was used to select independent risk factors for EPE among the significant variables on univariate analysis. The selected clinicopathological variables were then integrated with EPE grade evaluated by Reader 1 using a logistic regression method to build the Clinical + EPE grade model. To analyze the additional value of MRI to clinical models, we also built a MSKCCn + EPE grade model and a CAPRA + EPE grade model by using the same method.

The receiver operating characteristic (ROC) curves of the different models were plotted, and the AUC, diagnostic sensitivity, specificity, positive predictive value (PPV), and negative predictive value (NPV) were calculated to evaluate the diagnostic performance of these models. The DeLong test was used to compare the AUCs of the different models. For EPE grade, a predefined cut-off value (EPE grade ≥ 1) (15) was used, and for the other models, the Youden *J* index was used to determine the optimal cut-off (19). The sensitivities and specificities of these methods were compared by using the McNemar test.

The calibration curve together with the Hosmer-Lemeshow test were used to analyze the calibration performance of these models. The decision curves were also plotted to compare the clinical utility of these models. The software used for analyses included SPSS 22.0 (IBM), MedCalc 11.4.2.0 (MedCalc), and R 3.5.1 (Comprehensive R Archive Network, www.r-project.org). A two-tailed *P* value < 0.05 was indicative of statistical significance.

## Results

### Patient Demographic Characteristics

A total of 130 patients (mean age, 64.21 ± 8.10 years; range, 24–81 years) were included. Their median PSA level was 9.95 (2.78-83.02) ng/mL, median prostate volume was 34 (15-145) cm^3^, and the median PSAD was 0.31 (0.05–2.77). Pathologic EPE was diagnosed in 48.5% (63/130) of the patients. The clinicopathological characteristics of the patients included in this study are presented in **Table 1**.

**Table 1:** Clinicopathological characteristics of patients in this study (n = 130).

Variable	Value
Age (y)^*^	65 (24-81)
Prostate-specific antigen (ng/mL)^*^	9.95 (2.78-83.02)
Prostate volume at MRI (cm^3^)^*^	34 (15-145)
PSAD^*^	0.31 (0.05-2.77)
PI-RADS category	
2	4 (3.1)
3	9 (6.9)
4	58 (44.6)
5	59 (45.4)
Tumor location at MRI	
Anterior	63 (48.5)
Posterior	62 (47.7)
Diffuse	5 (3.8)
Percentage of positive biopsy cores^*^	0.33 (0.07-1.00)
ISUP category at biopsy	
1	42 (32.3)
2	35 (26.9)
3	28 (21.5)
4	9 (6.9)
5	16 (12.3)
cT stage	
1c	6 (4.6)
2	103 (79.2)
3	21 (16.2)
D’Amico risk group	
Low	12 (9.2)
Intermediate	28 (21.5)
High/locally advanced	90 (69.3)
Pathologic EPE	
Present	63 (48.5)
Absent	67 (51.5)
pT stage	
2	65 (50.0)
3a	55 (42.3)
3b	10 (7.7)

Unless otherwise indicated, data are numbers of patients, and data in parentheses are percentages. PSAD, prostate-specific antigen density; PI-RADS, Prostate Imaging Reporting and Data System; ISUP, International Society of Urological Pathology; EPE, extraprostatic extension. ^*^Data are the median (range).

### Univariate and Multivariate Analysis of Patients’ Clinicopathological Variables

In univariate analysis, PSA, PSAD, PI-RADS category, percentage of positive biopsy cores, ISUP category at biopsy, cT stage and D’Amico risk group were significantly different between the EPE positive and negative groups (all *P* < 0.05) (**Table 2**). No statistical significance was noted for age and tumor location (*P* > 0.05). After calculating the multicollinearity of these independents, the result showed that the variance inflation factors (VIFs) were < 10 (1.281-3.778), and tolerances were > 0.1 (0.265-0.781), which indicated that there was no potential collinearity problem. A forward stepwise logistic regression was used to select significant indicators among these variables, and PSAD and PI-RADS category were the independent risk factors for EPE (*P* = 0.007 and < 0.001, respectively). The two selected variables were then integrated with EPE grade to build the Clinical + EPE grade model.

**Table 2:** Univariate and multivariate analysis of patients’ clinicopathological variables.

Variable	β	Exp (β)	95% CI	*P*
Univariate analysis				
Age (y)	–	–	–	0.058
Prostate-specific antigen (ng/mL)	–	–	–	0.002
Prostate volume at MRI (cm^3^)	–	–	–	0.942
PSAD	–	–	–	0.007
PI-RADS category	–	–	–	< 0.001
Tumor location at MRI	–	–	–	0.274
Percentage of positive biopsy cores	–	–	–	0.002
ISUP category at biopsy	–	–	–	0.029
cT stage	–	–	–	0.002
D’Amico risk group	–	–	–	0.025
Logistic regression of clinicopathologic variables				
PSAD	1.561	4.765	1.520-14.945	0.007
PI-RADS category	1.297	3.660	1.947-6.881	< 0.001

CI, confidence interval; PSAD, prostate-specific antigen density; PI-RADS, Prostate Imaging Reporting and Data System; ISUP, International Society of Urological Pathology.

### Diagnostic Performance of the EPE Grade and Comparison With Clinical Models

**Figure 2:**
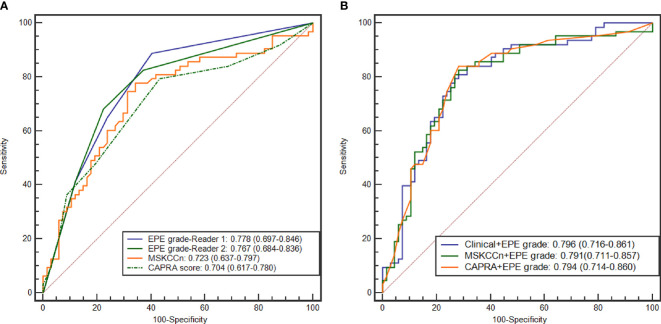
ROC curves of the EPE grade, MSKCCn, and CAPRA score **(A)**, and the Clinical + EPE grade model, MSKCCn + EPE grade model, and CAPRA + EPE grade model **(B)** for diagnosing EPE. ROC, receiver operating characteristic; EPE, extraprostatic extension; MSKCCn, Memorial Sloan Kettering Cancer Center nomogram; CAPRA, Cancer of the Prostate Risk Assessment.

The diagnostic performance according to the CAPRA score, MSKCCn, and EPE grade are presented in **Table 3** and **Figure 2**. The AUC for diagnosing EPE was 0.778 (95% confidence interval [CI]: 0.697–0.846) for EPE grade for Reader 1, 0.767 (95% CI: 0.684–0.836) for Reader 2, 0.704 (95% CI: 0.617–0.780) for the CAPRA score, and 0.723 (95% CI: 0.637–0.797) for MSKCCn. The AUCs of EPE grade, CAPRA score and MSKCCn were comparable (*P* > 0.05 for each comparison). The specificity of MSKCCn was significantly higher than the CAPRA score (65.7% vs. 56.7%, *P* = 0.031) and was comparable with the EPE grade (65.7% vs. 59.7%, *P* > 0.05). No significant difference was noted in sensitivity (88.9% for EPE grade in Reader 1, 79.4% for CAPRA, and 77.8% for MSKCCn, *P* > 0.05 for each comparison). The EPE grade for Reader 1 reached the highest NPV (85.1%) than other models. The intra- and interreader agreement of the EPE grade were both excellent, with a weighted kappa value of 0.805 and 0.877, respectively.

**Table 3:** Diagnostic performance of EPE grade, CAPRA score, MSKCCn, and combined model for extraprostatic extension.

	AUC	Sensitivity (%)	Specificity (%)	PPV (%)	NPV (%)
EPE grade-Reader 1	0.778 (0.697–0.846)	88.9 (78.4–95.4)	59.7 (47.0–71.5)	67.5 (60.5–73.8)	85.1 (73.4–92.2)
EPE grade-Reader 2	0.767 (0.684–0.836)	82.5 (70.9–90.9)	62.7 (50.0–74.2)	67.5 (59.9–74.3)	79.2 (68.4–87.1)
CAPRA	0.704 (0.617–0.780)	79.4 (67.3–88.5)	56.7 (44.0–68.8)	63.3 (56.0–70.0)	74.5 (63.3–83.2)
MSKCCn	0.723 (0.637–0.797)	77.8 (65.5–87.3)	65.7 (53.1–76.8)	68.1 (59.9–75.3)	75.9 (65.7–83.7)
Clinical + EPE grade	0.796 (0.716–0.861)	84.1 (72.7–92.1)	68.7 (56.2–79.4)	71.6 (63.5-78.5)	82.1 (71.8–89.3)
CAPRA + EPE grade	0.794 (0.714–0.860)	84.1 (72.7–92.1)	71.6 (59.3–82.0)	73.6 (65.3–80.6)	82.8 (72.7–89.6)
MSKCCn + EPE grade	0.791 (0.711–0.857)	82.5 (70.9–90.9)	71.6 (59.3–82.0)	73.2 (64.8–80.3)	81.4 (71.4–88.4)

AUC, area under the receiver operating characteristic curve; PPV, positive predictive value; NPV, negative predictive value; EPE, extraprostatic extension; CAPRA, Cancer of the Prostate Risk Assessment; MSKCCn, Memorial Sloan-Kettering Cancer Center nomogram.

### Diagnostic Performance of the Three Combined Models

The AUCs of the different combined models were comparable (*P* > 0.05 for each pair), and the values were 0.796 (95% CI: 0.716–0.861) for the Clinical + EPE grade model, 0.794 (95% CI: 0.714–0.860) for the CAPRA + EPE grade model, and 0.791 (95% CI: 0.711–0.857) for the MSKCCn + EPE grade model (**Table 3** and **Figure 2**). Compared with using clinical models alone, the combination of EPE grade significantly improved their diagnostic performance (CAPRA vs. CAPRA + EPE grade, *P* = 0.014; MSKCCn vs. MSKCCn + EPE grade, *P* = 0.027). Nevertheless, there was no statistically significant difference between the three combined models and EPE grade by itself (all *P* > 0.05). CAPRA + EPE grade showed improved specificity over the CAPRA score (71.6% vs. 56.7%, *P* = 0.041), and the Clinical + EPE grade model showed improved specificity over the EPE grade (68.7% vs. 59.7%, *P* = 0.031). There was no statistically significant difference in sensitivity among the EPE grade, clinical models, and the three combined models (all *P* > 0.05).

### Calibration Curves and Decision Curves of EPE Grade and Clinical Models

**Figure 3:**
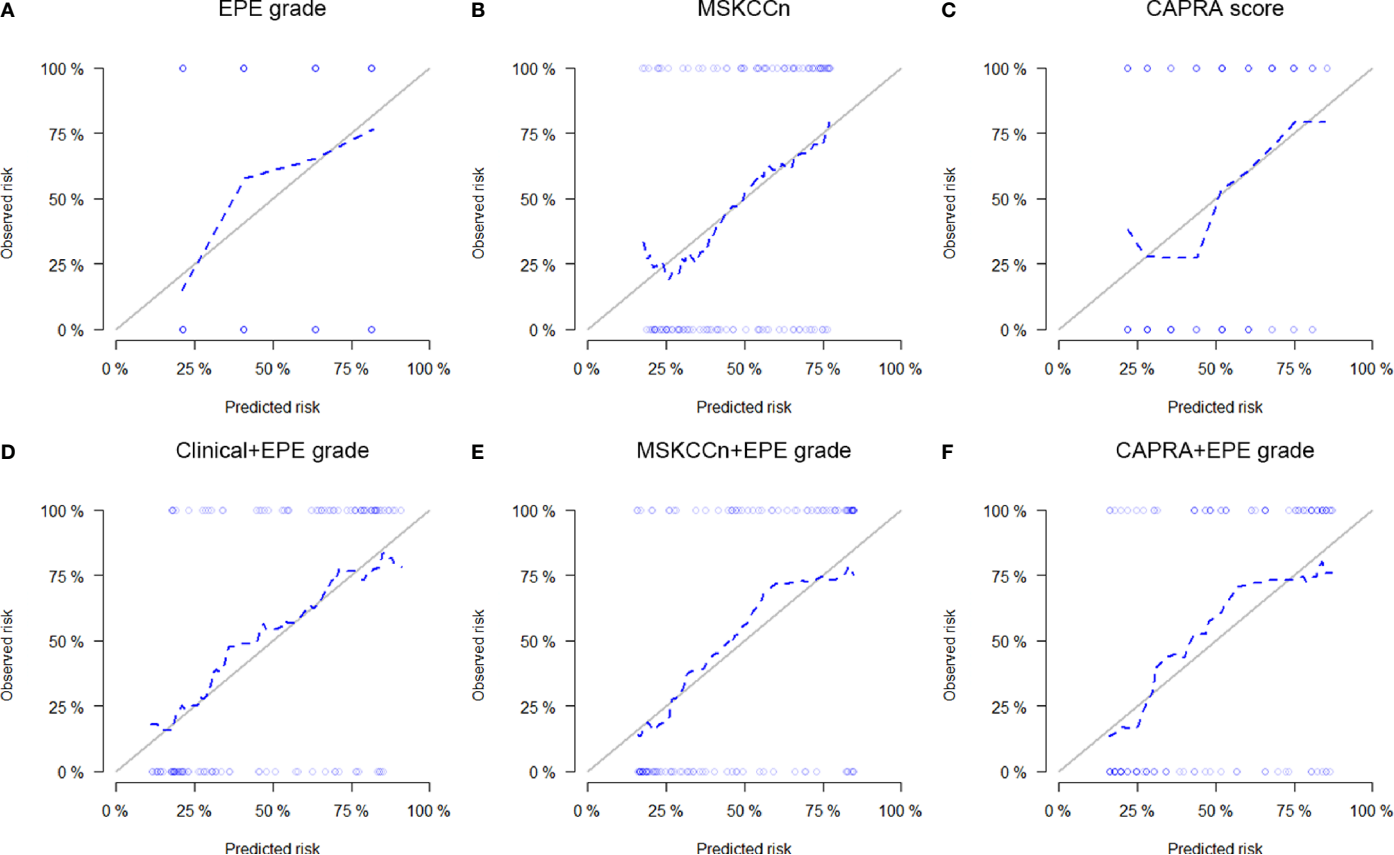
Calibration curves of the EPE grade **(A)**, MSKCCn **(B)**, CAPRA score **(C)**, Clinical + EPE grade model **(D)**, MSKCCn + EPE grade model **(E)**, and CAPRA + EPE grade model **(F)** for evaluating EPE. The Clinical + EPE grade model showed a poor model fit with statistically significant Hosmer-Lemeshow test result (*P* = 0.037), while other models were well-calibrated for EPE with non-significant Hosmer-Lemeshow test results (all *P* > 0.05). EPE, extraprostatic extension; MSKCCn, Memorial Sloan Kettering Cancer Center nomogram; CAPRA, Cancer of the Prostate Risk Assessment.

The calibration curves of the EPE grade and clinical models showed that these models are well-calibrated for EPE (**Figure 3**), and the Hosmer-Lemeshow test yielded a non-significant statistic (*P* = 0.394 for EPE grade, 0.780 for MSKCCn, and 0.281 for CAPRA). For the Clinical + EPE grade model, its Hosmer-Lemeshow test was statistically significant (*P* = 0.037), which indicated a poor model fit. As shown in the calibration plot (**Figure 3D**), the Clinical + EPE grade model was likely to underestimate the predicted risk. For the other combined models, the Hosmer-Lemeshow test showed no significant difference (*P* = 0.271 for MSKCCn + EPE grade, and 0.516 for CAPRA + EPE grade).

**Figure 4:**
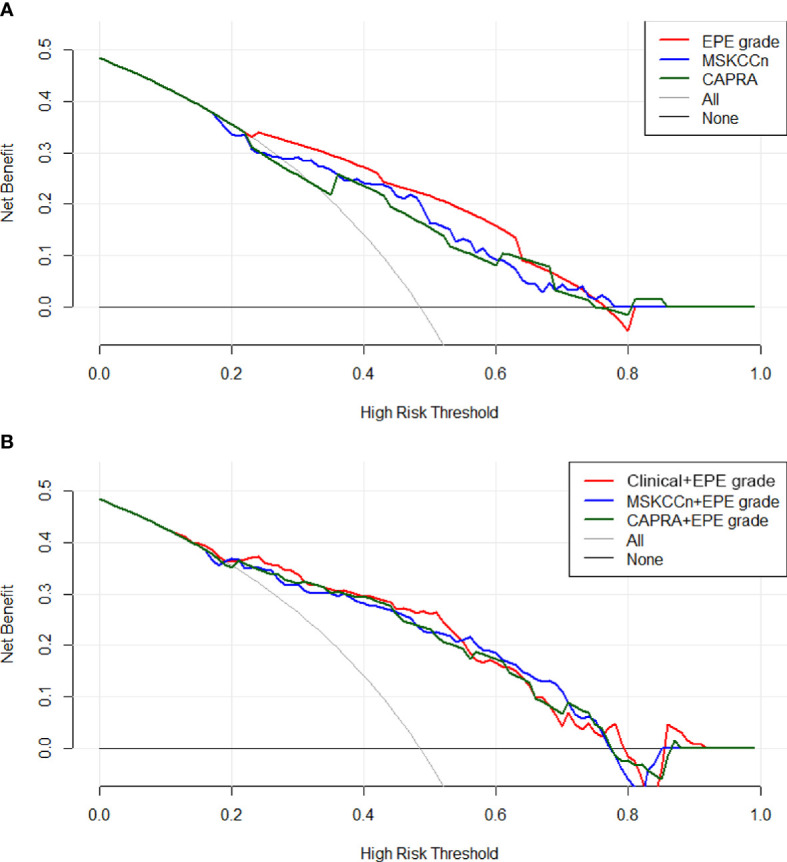
Decision curves of the EPE grade, MSKCCn, and CAPRA score **(A)**, and the Clinical + EPE grade model, MSKCCn + EPE grade model, and CAPRA + EPE grade model **(B)** for diagnosing EPE. The EPE grade showed a higher net benefit than the MSKCCn and CAPRA score, and the three combined models showed comparable net benefits. EPE, extraprostatic extension; MSKCCn, Memorial Sloan Kettering Cancer Center nomogram; CAPRA, Cancer of the Prostate Risk Assessment.

In the decision curve analysis, EPE grade showed slightly higher net benefit than the MSKCCn and CAPRA score (**Figure 4A**), and the three combined models showed comparable net benefits (**Figure 4B**).

## Discussion

In this study, we externally validated the EPE grade on MRI and compared this grading system with existing clinical models, MSKCCn and the CAPRA score. The EPE grade possessed good and comparable diagnostic performance with the clinical models for assessing EPE, with excellent inter- and intrareader agreements and clinical utility. Besides, combining the EPE grade with clinical models improved their diagnostic performance.

Compared with previous MRI grading methods, EPE grade provided a standardized and simplified grading system for EPE detection. This grading system is based on only a few imaging features, making it easy to teach and learn (14). In our study, by using EPE grade, even the junior radiologist could perform the EPE evaluation with a good diagnostic performance, which to some extent reflected the simplicity and comprehensibility of the EPE grade. Reisæter et al. compared the EPE grade with a five-point Likert score for EPE and the prediction of biochemical recurrence-free survival, and the results showed that the EPE grade and the EPE Likert have an equivalent diagnostic performance with a similar degree of observer dependence (20). Park et al. (15) compared the diagnostic performance of MRI-based criteria (including EPE grade, European Society of Urogenital Radiology score, Likert scale, and capsular contact length) for the assessment of EPE, and these criteria showed good overall diagnostic performance, with AUC ranges of 0.77–0.81, 0.79–0.81, 0.78–0.79, and 0.78–0.85, respectively, with substantial intra- and interreader agreement. Further analysis showed that EPE grade had the highest correlation with histologic extent of EPE, and in this regard, the EPE grade resulted in a more reliable performance (15). In our study, we compared EPE grade with existing clinical models and found the EPE grade showed comparable diagnostic performance with them. Regarding the clinical utility of these models, EPE grade might be more helpful than MSKCCn and the CAPRA score. The good performance of the EPE grade may lie in the integrating of quantitative (curvilinear contact length) and qualitative variables (capsular bulge and frank EPE). In a recently published meta-analysis including thirteen articles with 2136 patients, the diagnostic performance of tumor capsular contact was good with a summary sensitivity and specificity of 0.79 and 0.67, respectively, and the AUC was 0.81 (21). Additionally, an important information provided by EPE grade and not offered by the clinical nomograms is EPE location which should help tailor surgical approach and potentially reduce margin positivity rates.

Regarding individualized treatment, there is a need to integrate clinical risk factors with MRI imaging features to more accurately predict the possibility of EPE (22, 23). Studies have shown that MRI features can improve the diagnostic performance of clinical-based models to predict EPE (11, 12). Morlacco et al. analyzed the diagnostic performance of using PT and CAPRA score alone, and with the application of MRI for detecting EPE, the AUC was 0.61 vs. 0.73 (without and with MRI) for PT and was 0.69 vs. 0.77 (without and with MRI) for the CAPRA score (11). In Rayn et al.’s research, the AUC was 0.78 for MRI, 0.70 for MSKCCn, and 0.66 for PT, and the AUC increased after combining with MRI and was 0.80 (*P* = 0.003) for MRI + MSKCCn and 0.80 (*P* < 0.001) for MRI + PT. In another study based on 73 PCa patients that aimed to compare the mpMRI, PT, MSKCCn, and CAPRA score in predicting EPE, only the combination of MRI with CAPRA provided a significantly higher AUC than using CAPRA alone (24). This trend can also be found in our study. After combining with EPE grade, the diagnostic performance of MSKCCn + EPE grade, and CAPRA + EPE grade increased significantly relative to using MSKCCn and the CAPRA score alone. The combination of the EPE grade increased both the PPV and NPV of these clinical models, which means that compared with using clinical models alone, take the EPE grade into account would be helpful for tumor control as well as preserve patients’ function. Besides, we also combined the PSAD and PI-RADS category with EPE grade to build our own combined model. The Clinical + EPE grade model showed a higher AUC than the other clinical models, but the calibration ability of the combined model is poor. Therefore, further improvement of this model is needed for individualized risk prediction.

MRI is a well-recognized method to improve clinical-based models’ performance in the prediction of EPE; nevertheless, a few studies have reported the incremental value of clinical variables to MRI criteria. Martini et al. (25) developed a side-specific predictive model based on clinical variables and MRI for EPE. The model’s AUC was higher than MRI (82.92% vs. 68.83%, not statistically demonstrated). However, in this study, EPE on mpMRI was a binary variable, which may not be suitable for EPE evaluation in clinical practice, since it is generally acknowledged that interpretations should estimate the likelihood of pathologic EPE (14). In our research, the combination of clinical models and variables to EPE grade just showed comparable diagnostic performance to using MRI criteria alone. Compared with the EPE grade, these combined models showed decreased NPV but increased PPV, this would benefit patients from receiving nerve-sparing surgery, but increase their risk of positive surgical margins and opportunity of post-surgery treatments. Compared with clinicopathological variables, MRI could provide more visible information for EPE evaluation, and thus it might be reasonable to assume that its performance is less likely to be affected by patient cohort differences. Apart from integrating clinical factors to increase the performance of EPE grade, risk stratification is another way worth trying to make this grading system more useful in patient management and decision making (26–28).

There are several limitations to our study. First, it was a retrospective single-center study, and prospective multi-center studies are needed to evaluate the effect of EPE grade in personalized decision making. Second, the diagnostic performance for side-specific EPE statues was not reported as previous research (29), since this study aimed to compare the EPE grade with existing and thoroughly investigated clinical nomograms which were not used for side-specific purpose. A thorough and direct comparison of per-lesion EPE grade and pathologic results will be conducted in our future studies. Finally, we have not analyzed the relationship of EPE grade with surgical margin status, which would be helpful for urologists to mitigate the occurrence of positive surgical margins.

In conclusion, the EPE grade showed good and comparable performance with clinical models for evaluating EPE with well clinical utility and excellent inter- and intrareader agreements. Additionally, combination with the EPE grade could improve the diagnostic performance of clinical models.

## Data Availability Statement

The original contributions presented in the study are included in the article/**Supplementary Material**. Further inquiries can be directed to the corresponding authors.

## Ethics Statement

The studies involving human participants were reviewed and approved by Institutional Review Board of Peking Union Medical College Hospital. Written informed consent for participation was not required for this study in accordance with the national legislation and the institutional requirements.

## Author Contributions

Guarantor of the article: ZJ and HS. Conception and design: HS and LX. Collection and assembly of data: GZ, LX, XZ, and XB. Data analysis and interpretation: LX, GZ, WY, YX, and HS. All authors contributed to the article and approved the submitted version.

## Funding

This study has received funding by the National Natural Science Foundation of China (grant no. 91859119), the Non-profit Central Research Institute Fund of Chinese Academy of Medical Sciences (grant no. 2019XK320028), the National Natural Science Foundation of China (grant no. 81901742), the Natural Science Foundation of Beijing Municipality (grant no. 7192176), the Central University Basic Scientific Research Business Expenses Special Funds (grant no. 3332018022), the National Public Welfare Basic Scientific Research Project of Chinese Academy of Medical Sciences (grant nos. 2019PT320008 and 2018PT32003). All the funding supported equally in the design of the study and collection, analysis, and interpretation of data and in writing the manuscript.

## Conflict of Interest

The authors declare that the research was conducted in the absence of any commercial or financial relationships that could be construed as a potential conflict of interest.
