# Supplementary material for: External Validation of the Extraprostatic Extension Grade on MRI and Its Incremental Value to Clinical Models for Assessing Extraprostatic Cancer
Source: Front Oncol. 2021 Apr 1;11:655093. doi: 10.3389/fonc.2021.655093 (PMC8047629; doi:10.3389/fonc.2021.655093)
Supplement: Supplementary file 1 [file Table_1.doc]

Supplementary Material

**1 Supplementary Table 1. Prostate multiparametric magnetic resonance imaging protocol**

| Parameters | T2WI | DWI | DCE |
| --- | --- | --- | --- |
| Sequence | FRFSE | SE-EPI | 3D-GRE |
| TR/TE (ms) | 4137/86 | 4200/90 | 4.3/1.3 |
| Flip angle (degree) | 110 | 90 | 12 |
| Echo train length | 32 | 1 | N/A |
| Field of view (mm × mm) | 270 × 270 | 360 × 360 | 400 × 400 |
| Matrix size | 288 × 192 | 128 × 96 | 320 × 192 |
| Thickness (mm) | 3.0 | 3.0 | 3.0 |
| Other |  | b values = 0, 100, 150, 200, 500, 800, 1000, 1500, 2000 mm^2^/sec | Temporal resolution <10s, and total scan time of 5 min |

*T2WI, T2-weighted imaging; DWI, diffusion-weighted imaging; DCE, dynamic contrast-enhanced; TR, repetition time; TE, time echo; FRFSE, fast relaxation fast spin echo; SE-EPI, spin-echo echo planar imaging; 3D-GRE, 3D-gradient echo.*
